# Identification of Carbohydrate Metabolism Genes in the Metagenome of a Marine Biofilm Community Shown to Be Dominated by Gammaproteobacteria, Bacteroidetes

**DOI:** 10.3390/genes1030371

**Published:** 2010-10-26

**Authors:** Jennifer L. Edwards, Darren L. Smith, John Connolly, James E. McDonald, Michael J. Cox, Ian Joint, Clive Edwards, Alan J. McCarthy

**Affiliations:** 1 Microbiology Research Group, School of Biological Sciences, BioSciences Building, University of Liverpool, Crown Street, Liverpool, L69 7ZB, UK; E-Mails: jenedw@live.co.uk (J.L.E.), smithdx@liverpool.ac.uk (D.L.S.), john76@liverpool.ac.uk (J.C.), j.mcdonald@bangor.ac.uk (J.E.M.), mikeyj.cox@gmail.com (M.J.C.), kj26@liverpool.ac.uk (C.E.); 2 Plymouth Marine Laboratory, Prospect Place, The Hoe, Plymouth, PL1 3DH, UK;E-Mail: irj@pml.ac.uk

**Keywords:** 454 pyrosequencing, next generation sequencing, marine polysaccharide degradation, glycoside hydrolases, metagenomics, marine bacteria, cellulose biofilm

## Abstract

Polysaccharides are an important source of organic carbon in the marine environment, degradation of the insoluble, globally abundant cellulose is a major component of the marine carbon cycle. Although a number of species of cultured bacteria are known to degrade crystalline cellulose, little is known of the polysaccharide hydrolases expressed by cellulose-degrading microbial communities, particularly in the marine environment. Next generation 454 Pyrosequencing was applied to analyze the microbial community that colonizes, degrades insoluble polysaccharides *in situ* in the Irish Sea. The bioinformatics tool MG-RAST was used to examine the randomly sampled data for taxonomic markers, functional genes,, showed that the community was dominated by members of the *Gammaproteobacteria, Bacteroidetes*. Furthermore, the identification of 211 gene sequences matched to a custom-made database comprising the members of nine glycoside hydrolase families revealed an extensive repertoire of functional genes predicted to be involved in cellulose utilization. This demonstrates that the use of an *in situ* cellulose baiting method yielded a marine microbial metagenome considerably enriched in functional genes involved in polysaccharide degradation. The research reported here is the first designed to specifically address the bacterial communities that colonize, degrade cellulose in the marine environment, to evaluate the glycoside hydrolase (cellulase, chitinase) gene repertoire of that community, in the absence of the biases associated with PCR-based molecular techniques.

## 1. Introduction 

It is established that bacteria are mainly responsible for the consumption of organic matter in the marine environment,, the carbon cycle is largely dependent upon this remineralization of biomass. The polysaccharides, proteins, lipids produced as a result of carbon fixation together form the core of the particulate organic matter (POM), dissolved organic matter (DOM) pools in the marine environment. POM, DOM are mineralized, oxidized by heterotrophic marine bacteria to generate microbial biomass, inorganic products [1]. Biogeochemical processes within the marine environment as a whole are dynamic, complex,, are poorly understood [2]. Furthermore, microbial community structure varies significantly between coastal, open waters, at different water depths,, also seasonally. Through 16S rRNA gene sequence analysis, marine microbial populations have been cataloged to show that particle-attached, free-living communities differ, with the former colonized by bacteria (related to Cytophaga spp, Planctomyces spp, the γ Proteobacteria), while the planktonic fraction is rich in members of the α Proteobacteria [3]. Whole genome shotgun sequencing (WGS) of filtered sea water from the Sargasso Sea also confirmed the predominance of Proteobacteria (primarily α, β, γ subgroups) followed by the Firmicutes, Cyanobacteria, Bacteroidetes when a range of phylogenetic markers were used for comparison [4]. More recently, metagenomic, metatranscriptomic analysis of ocean surface water has shown the Cyanobacteria (genus Prochlorococcus), Alphaproteobacteria (genus Roseobacter) to be the best represented taxonomic groups [5]. However, few studies have directly addressed structural polysaccharide degradation in marine ecosystems,, have focused entirely on the isolation, identification of bacterial species, including members of the genus Pseudoalteromonas. [6,7]. More recently, Saccharophagus degradans, Teredinibacter turnerae have emerged as strong examples of two well characterized marine bacterial species involved in polysaccharide degradation,, whole genome annotations have revealed an extensive repertoire of relevant functional genes [8,9]. There is a diversity of polysaccharide structures, sources in the marine environment, but cellulose is well represented, likely to persist in POM due to the recalcitrance imparted by its crystalline structure. 

The Glycoside Hydrolases (GHs) are modular enzymes that hydrolyse glycosidic bonds of carbohydrates, with classification based on amino acid sequence, predicted three-dimensional structure. Such enzymes may contain single or multiple catalytic modules (GH) together with single or multiple non-catalytic carbohydrate-binding modules (CBMs) [10,11,12]. While the screening of large insert, vector based metagenomic libraries from other environments has resulted in the occasional identification of cellulase genes, these have typically been present at very low frequencies. Only four cellulase positive clones were identified amongst ca. 100,000 cosmids prepared from a sample of compost DNA [13], 70,000 clones of ~40 kbp constructed from soil DNA yielded only one cellulase positive clone [14]. These data suggest that fosmid, cosmid-derived metagenomic libraries of environmental samples do not provide an adequate assessment of the functional diversity of GH genes, even for microbial communities that would be expected to retain a significant capability for polysaccharide biodegradation. Extraction of DNA fragments of adequate size from environmental samples is a limiting step in large insert library based metagenomics, whereas 454 pyrosequencing is a high throughput alternative to identifying functional genes present in microbial communities from relatively short fragments of DNA. A number of studies have successfully targeted environments known to be rich in polysaccharide-degrading microorganisms, including the hindgut microbiota of a wood feeding termite, bovine rumen contents, an enriched switchgrass composting system [15-17]. 

The majority of marine bacteria have yet to be isolated in culture, resulting in a paucity of information on community structure, function, particularly for colonized organic matter representing the primary stage of carbon mineralization in the sea. Metagenomic studies offer the potential to gain an unprecedented insight into such communities but, as has been demonstrated in previous DNA sequencing projects [4], marine microbial communities are much too diverse to generate sequence information that provides adequate coverage of the total community. In order to circumvent the problem of under-representation of genes for environmentally important primary functions, we have employed an in situ enrichment technique previously used to both identify, isolate cellulolytic organisms in freshwater lakes [18,19]. Here, in situ colonized cellulose ‘bait’ is used as the source of biological material for metagenomic studies directed at organisms, genes involved in this primary step in carbon recycling in the Irish Sea.

## 2. Results, Discussion 

### 2.1. Metagenome Analysis

Cellulose baits were tethered to a Cefas SMART buoy in the Irish Sea,, recovered after one month in situ incubation. Total DNA was extracted from the microbial biofilm that colonized the cotton string, 454 pyrosequencing was performed on the community DNA. This metagenomic sequencing generated 223,263 reads of DNA sequence, containing 48,338,140 bp of DNA with a size range of 8-375 bp. The raw read data were assembled into 26,860 contiguous sequences (contigs) with a size range of 93-26,859 bp, comprising 6,841,343 bp in total. Of the assembled contigs in the dataset, the majority were less than 1 kb in length, suggesting significant heterogeneity within the sample. One contig of ~26 Kb was removed from the dataset, analysed separately as it was identified to have sequence similarity with a bacteriophage (data not shown). The phylogenetic diversity within the dataset was assessed using the SEED MG-RAST (Metagenome Rapid Annotation Using Subsystem Technology) [20]. Taxonomic information on the sequences within the metagenomic data was obtained by comparing the contiguous sequences against the Greengenes 16S rRNA gene database [21]. Using an expected cut off (e-value) of 1 × 10-5, a minimum alignment of 50 bp, a total of 18 partial 16S rRNA gene fragments were identified. Fourteen of these were assigned to the Bacteria, of which four are affiliated with the phylum Bacteroidetes (Flavobacterium; Ulvibacter; Roseivirga, unclassified Cytophagaceae), ten with the phylum Proteobacteria (Sulfitobacter; unclassified Rhodobacteraceae (3); Glaciecola (3); Teredinibacter; Cellvibrio, unclassified Gammaproteobacteria) (Table 1). The remaining four contigs identified as fragments of 16S rRNA genes could not be assigned. The occurrence of 16S rRNA genes in the pyrosequenced dataset reported was therefore one in every 1,413 contigs. 16S rRNA gene-based phylogenetic studies have become routine in microbial ecology,, through such studies it is recognized that Bacteroidetes (particularly members of the genus Cytophaga), members of the alpha, gamma lineages of the Proteobacteria colonize, contribute to the mineralization of organic aggregates, while noting that the community structure of particle-attached, free-living bacteria differ [2,22-25]. Due to the inherent complexity of marine biofilms [26], it has become clear that recent metagenomic studies have barely scratched the surface of the total phylogenetic, functional diversity of the marine microbial community. There is a clear advantage in including an in situ enrichment step for capturing functional genes that are involved in influential processes in marine carbon cycling as well as the dominant bacterial groups residing within the polysaccharide colonizing community.

**Table 1:** Identification of 16S rRNA genes in the Irish Sea cellulose biofilm DNA 454 pyrosequenced dataset by the Greengenes 16S rRNA gene database.

Sequence ID	Alignment Length	E-value	% Identity	Bit Score	Fragment (Start - End)	Taxonomy Assignment	Best Hit ID
**Proteobacteria**	****	****	****	****	****	****	****
contig16635	85	1.00E-40	100	168	1 - 85	Sulfitobacter	165917
contig26360	150	2.51E-51	93	202	1 - 149	Unclassified Rhodobacteraceae	142124
contig26572	162	7.94E-70	96	264	4 - 163	Unclassified Rhodobacteraceae	70710
contig26707	130	2.51E-63	99	242	1 - 130	Unclassified Rhodobacteraceae	113926
contig25574	235	2.51E-130	100	466	50 - 284	Glaciecola	108683
contig26573	526	0.00E+00	99	1003	1 - 526	Glaciecola	80428
contig26860	151	1.26E-80	100	299	1 - 151	Glaciecola	170839
contig26820	696	0.00E+00	93	1015	6 - 701	Teredinibacter	144812
contig00070	247	1.26E-81	93	305	738 - 983	Cellvibrio	98921
contig12909	577	0.00E-00	93	858	209 - 784	Unclassified Gammaproteobacteria	151615
**Bacteriodetes**	****	****	****	****	****	****	****
contig00061	892	0.00E+00	90	1094	1988 - 2879	Roseivirga	102384
contig26228	139	2.51E-66	98	252	1 - 138	Flavobacterium	154970
contig26430	241	5.01E-130	99	464	1 - 241	Ulvibacter	80102
contig26765	436	5.01E-140	89	500	60 - 495	Unclassified Cytophagaceae	2577

Best Hit is the ProkMSA Greengenes reference ID.

**Figure 1:**
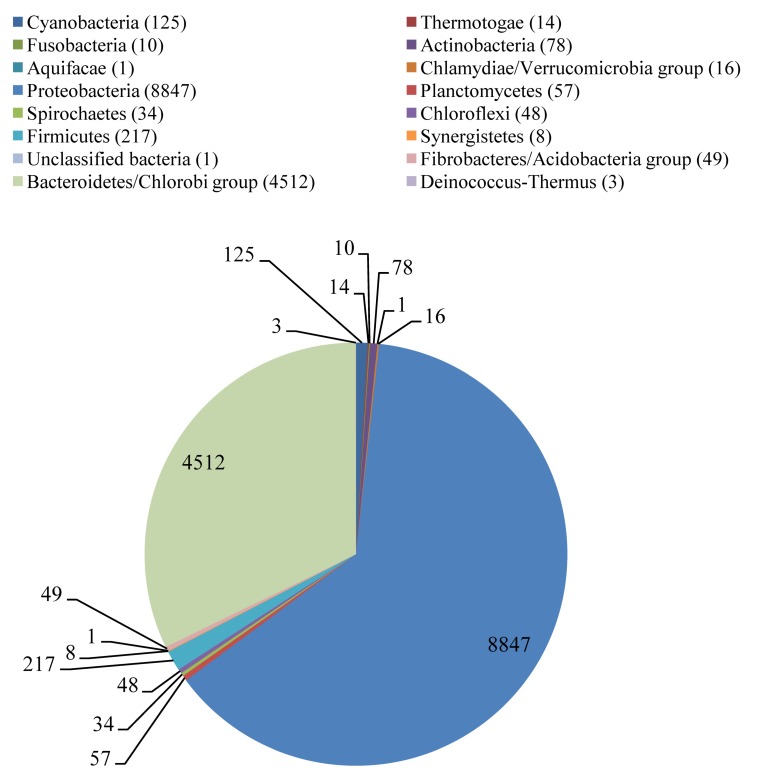
Summary of Phylum level taxonomic assignment of 14,020 assembled contigs matching protein encoding genes (PEGs).

**Figure 2:**
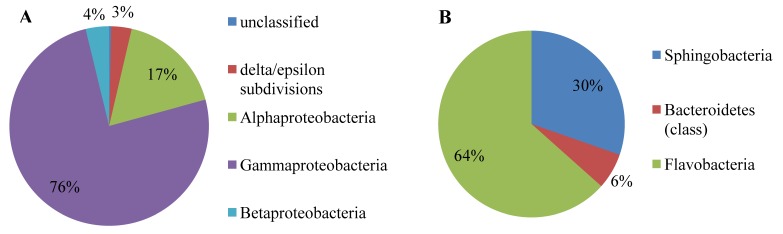
Taxonomic assignment of 13,312 sequence contigs matching Protein encoding genes (PEGs) of the Proteobacteria, the Bacteroidetes. The percentage of contigs matching PEGs assigned at the Class level using the SEED MG-RAST (Metagenome Rapid Annotation Using Subsystem Technology are shown for (A) Proteobacteria (8847; 61% of assignments to the Bacteria), (B) Bacteroidetes (4465; 32% of assignments to Bacteria). Values are shown as percentages of the total contigs assigned to each class within the two Phyla.

Further information on the taxonomic diversity residing within this Irish Sea metagenome dataset was obtained by investigating protein-encoding genes as taxonomic markers. This was achieved by comparing the assembled contigs against the SEED-nr database [27]. A total of 14,179 contigs could be assigned to known functional genes, of which 14,020 were to the Bacteria (98.9% of positive matches), with Eukaryota, Viruses, Archaea accounting for 0.65%, 0.18%, 0.29% of the positive hits, respectively. Taxonomic assignment of the biofilm community was dominated by members of the Proteobacteria (8847; 61% of the assignments to Bacteria), Bacteroidetes (4465; 32% of assignments to Bacteria) (Figure 1). Although only 14 contigs were identified as fragments of bacterial 16S rRNA genes, the markers of choice for taxonomic assignments, it is encouraging that the predominance of Proteobacteria, Bacteroidetes discovered therein (Table 1) is mirrored by the SEED MG-RAST analysis of the distribution of taxonomic assignments in over 14,000 contigs from protein coding genes (Figure 1) in which Gammaproteobacteria, Flavobacteria dominate the assignments at the class level (Figure 2). Notably the emergence of the carbohydrate degrading Microbulbifer/Teredinibacter/Saccharophagus group of the Gammaproteobacteria [28], their role in marine polysaccharide degradation is in keeping with the results found here. This is also true of the well established role of Bacteroidetes, formerly known as the Cytophaga/Flavobacteria/Bacteroides group,, their dominance in the marine environment particularly in coastal regions [29,30], their role in phytoplankton colonization [31], polysaccharide degradation [30]. The conclusion that this biofilm colonizing cellulose suspended in the Irish Sea is dominated by these two major bacterial groups can therefore be made with some confidence. 

### 2.2. Polysaccharide hydrolases 

Glycoside Hydrolase families containing representatives of selected functions such as endoglucanase, chitinase were identified, catalytic domains downloaded from the Pfam database. This customized database comprised a total of 14,332 protein sequences distributed across twelve of the GH families (5, 6, 7, 8, 9, 12, 16, 18, 19, 45, 48, 61). Comparison of the 26,859 metagenome pyrosequencing-derived contigs to the constructed GH database provided 211 hits at an E value restriction of 1 × 10-5 (Table 2; a complete list of all those contigs with matches to sequences in the constructed GH database is provided in Supplementary Table 1). The most frequently occurring GH families were GH5 (56; 27% of total matches), 8 (40; 19%), 9 (30; 14%), 16 (64; 30%). GH5 is one of the largest, most diverse of the GH families, with several known enzymatic functions. GH families 8, 9 primarily contain endoglucanases, cellobiohydrolases, whilst GH16 proteins show Endo-1,3(4)-β glucanase, xyloglucanase activities against carbohydrates that are usually intertwined with cellulose in plant cell wall material [10,32,33]. The contigs that were identified to have sequence similarity were then compared against the NCBI nr database using BLASTX [34]. Sequence similarity hits that were most frequent were proteins from the Gram negative, rod shaped aerobic Gammaproteobacterium S. degradans (27 matches), a marine cellulolytic bacterium, Teredinibacter turnerae [8,28] (17 matches), a Gammaproteobacterium closely related to S. degradans that occurs as an intracellular endosymbiont in the gills of wood boring bivalves [9], the Gram-negative, aerobic, rod-shaped gliding Bacteroidete Cytophaga hutchinsonii (48 matches), a cellulolytic bacterium originally isolated from soil [35]. Taken together, these account for 44% of all the hits to the GH database summarized in Table 2; a complete list of all hits against the NCBI nr database is provided in Supplementary Table 2). All three of these named species are known to be actively cellulolytic, containing a plethora of enzymes involved in the hydrolysis of polysaccharides [8,9,35]. 

**Table 2:** Summary of the number of 454 sequencing contigs matching each GH family.

CAZy family	Pfam ID	Number of hits
GH5	PF00150	56
GH6	PF01341	5
GH7	PF00840	0
GH8	PF01270	40
GH9	PF00759	30
GH12	PF01670	1
GH16	PF00722	64
GH18	PF00704	10
GH19	PF00182	3
GH45	PF02015	0
GH48	PF02011	2
GH61	PF03443	0

Searches were performed using Blastx for all 26,859 assembled contigs against the customized library of downloaded GH families using an E value cut-off of 1 × 10-5 Pfam ID is the protein family [36], CAZy (carbohydrate active enzymes database family [10]. A comprehensive list of all hits, values can be found in Supplementary Table 1.

The quantity of contigs providing sequence similarity to GH supports the use of in situ cellulose baits as a means of enriching the metagenome in genes that encode polysaccharide-degrading functions. Ninety–eight of the sequence matches to the constructed GH database are unique, whereby one contig matches one sequence in the database, whereas the remainder had sequence similarity with at least two other contiguous sequences (Supplementary Table 3). For example, 13 contigs matched a GH8 family protein from the Bacteroidete C. hutchinsonii (Q11PI8_CYTH3), nine further contigs also hit another GH8 family protein from C. hutchinsonii (Q11VQ4_CYTH3), nine contigs hit a GH16 family protein from the Gammaproteobacterium S. degradans (Q21KX4_SACD2). Only 10 (<10%) contigs matched proteins of GH family 18,, three contigs matched to GH19 proteins (Table 2), the two families that contain all known chitinases (http://www.cazy.org). Two contigs matched the same protein, C1S930_9SPHI, from the chitinolytic Bacteroidete, Chitinophaga pinensis. A few culture-independent molecular biological studies have addressed GHs in the marine environment, but these have focused mainly on chitinases, due to the fact that marine vibrios are often chitinolytic, traditionally amongst the most readily isolated, cultivated marine bacteria [37]. Cottrell et al. [38] screened metagenomic libraries of coastal, estuarine water DNA using fluorogenic analogues of chitin, cellulose to identify a number of genes involved in chitin hydrolysis, but none that expressed proteins with activity against the cellulose analogue. The diversity, abundance of β-1,4 endoglucanases within the GH5 family in the North Atlantic Ocean has been investigated by designing primers for a 350 bp fragment constituting ~one-third of the gene. Relative abundance was determined for three locations using qPCR,, found to positively correlate with chlorophyll concentrations. Analysis of clone libraries showed that the GH5 family genes were more diverse in samples from coastal water than those from the open ocean [39]. Cottrell et al. [40] screened a fosmid library constructed from prokaryotic DNA from the Western Arctic Ocean. PCR primer sets were designed for the most abundant type of endoglucanase identified in the Sargasso Sea WGS dataset [4], but subsequent functional analysis revealed that the gene encoded a peptidase. Functional molecular ecology studies targeting polysaccharide degradation in the marine environment are clearly fragmentary in nature. 

### 2.3. Scanning Electron Microscopy analysis of colonized cellulose bait

**Figure 3:**
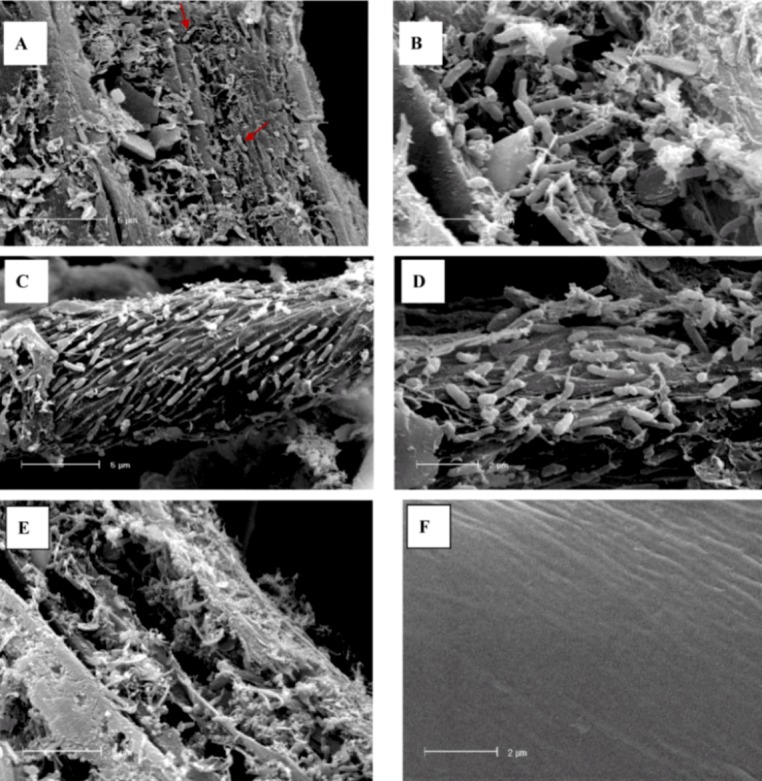
Scanning electron micrographs of the microbial community colonizing cellulose ‘bait’ in situ in the Irish Sea. (A) A cellulose fiber heavily colonized by a biofilm matrix. Rod-shaped bacteria can clearly be seen occupying grooves or pits (arrow) providing visible evidence of degradation by these cellulolytic bacteria; (B) a close image (bar = 2 µm) of a hollowed out region of a cellulose fiber with abundant rod-shaped bacteria; (C) rod-shaped bacteria arranged in rows around the cellulose surface with grooves visible where bacterial cells have formed pits on the surface; (D) a closer view of a cellulose fiber where rod shaped bacteria are located in grooves on the surface; (E) surface of cotton string heavily colonized by biofilm matrix showing extensive degradation of the cellulose surface, in comparison to (F) uncolonized cotton string.

The surface of colonized cellulose recovered from the same sampling site in the Irish Sea was visualized using SEM. The cellulose bait was heavily colonized (Figure 3 a-c, e), with predominantly rod shaped bacteria (Figure 3 c, d). Microorganisms were often arranged in rows on the cellulose surface (Figure 3c) with pockets visible where degradation had occurred (Figure 3). In areas of heavy colonization by the dense biofilm matrix, visible signs of degradation were observed where the cotton surface had been eroded (Figure 3 a, b). In comparison the surface of cotton string not colonized by microorganisms is smooth in appearance (Figure 3f). Protuberances are apparent on the surface of many rod-shaped cells (Figure 3d) one explanation for which could be the presence of polycellulosomes, the macromolecular structures responsible for cellulase activity in a number of bacterial species [41]. S. degradans, the marine aerobic polysaccharide-degrading species whose proteins gave matches to over 10% of the GH hits above, produces surface structures in the presence of cellulose [28],, a number of hydrolytic enzymes are thought to be consorted in such S. degradans complexes [8]. It has been suggested through genome sequence analysis of S. degradans, T. turnerae that Gram-negative bacteria use lipoproteins to anchor carbohydrate active enzymes to the outer membrane, playing a similar role to that of cellulosomes that are usually associated with Gram-positive bacteria [8,9]. S. degradans, C. hutchinsonii which both make up a large proportion of hits to GHs are known to be Gram-negative aerobic rod-shaped bacteria [28,34]. The morphology of which is in keeping with that seen in the SEM pictures (Figure 3 a-e).

## 3. Experimental Section 

### 3.1. Sampling 

Cotton yarn (0.7 cm diameter) (Lancashire cotton best twist, from Texere Yarns, Bradford, UK) was used as the cellulose bait. Approximately 1 m of yarn was placed in customized nylon mesh bags (10 cm × 10 cm). ‘Baits’ were tethered to a Cefas SMART (http://www.cefas.co.uk) buoy in the Eastern Irish Sea in surface water (53º 27´ N 3º 38.6´ W) for a period of 1 month. 

### 3.2. Metagenome Analysis

#### 3.2.1. DNA extraction [42]

Nucleic acids were extracted by placing 0.5 g (wet weight) string (retrieved from the Irish Sea following one month in situ, April, 2008) in a Q-biogene purple top multimix tube (lysing matrix E). 0.5 ml hexadecyltrimethylammonium bromide (CTAB) buffer [prepared by mixing equal volumes of 10 % (w/v) CTAB in 0.7 M NaCl with 240 mM potassium phosphate buffer, pH 8.0] was added along with phenol-chlorofom-isoamyl alcohol (25:24:1; pH 8.0). Cells were lysed by bead beating in a Ribolyser for 30 s at a speed of 5.5 m/s,, the aqueous phase containing nucleic acids separated by centrifugation (16,000 × g) for 5 min at 4 oC. The aqueous phase was transferred to a fresh microfuge tube, phenol removed by mixing an equal volume of chloroform-isoamyl alcohol (24:1), followed by centrifugation at (16, 000 × g) for 5 min. Nucleic acids were obtained by precipitation of the top layer by the addition of 2 volumes of 30% polyethylene glycol (PEG) solution (30% polyethylene glycol, 1.6 M NaCl), incubated overnight at 4 oC. The precipitated nucleic acids were collected by centrifugation (16, 000 × g) for 15 min. The supernatant was removed, the pellet washed with 200 μl 70% ice cold ethanol, air dried prior to resuspension in 50 μl sterile ddH2O.

#### 3.2.2. Pyrosequencing, sequence analysis

DNA was sequenced using the 454 Corporation’s GS-FLX instrument at the NERC-funded Advanced Genomics Facility at the University of Liverpool (http://www.liv.ac.uk/agf/). 26,859 assembled contigs were uploaded in a FastA format to the MG-RAST server at the SEED [20,43] on 15 February 2010 under the name Irish_Sea_Metagenome, was assigned the Metagenome ID: 4446437.3.

#### 3.2.3. Glycoside Hydrolase database construction

The GHs representing classes of GHs of functional interest (e.g. endoglucanase, chitinase) were identified by analyzing the GH families in the Carbohydrate Active Enzyme (CAZy) web resource. All of the protein sequences of catalytic domains of GH families 5, 6, 7, 8, 9, 12, 16, 18, 19, 45, 48, 61 were downloaded (February 2010) from the Pfam database [36]. These families were chosen based on known functions, including mainly cellulase, chitinase ability (families18, 19). All metagenome-derived contigs were used as a query, compared against the GH database using Blastx. Only those hits with an E value cut-off of 1 × 10-5 were recorded.

### 3.3. Scanning Electron Microscopy (SEM) of colonized cellulose samples

Samples of cellulose bait were collected from the mooring site in the Irish Sea (January, 2009), refrigerated during transport to the laboratory. The samples were gently rinsed with ddH2O, immersed in excess absolute Ethanol (Sigma) which had been pre cooled to -80 oC,, the samples returned to -80 oC overnight. The samples were then removed, placed into a universal bottle containing pre-cooled absolute ethanol,, again returned to -80 oC until required. Specimens were dried from absolute ethanol in carbon dioxide using a Polaron E3000 critical point dryer, glued to stubs, sputter-coated with 60 % gold-palladium in a Polaron E 5100 coater, viewed in a Philips 501B scanning electron microscope at accelerating voltages of 7.2, 15 kV [44]. Final sample preparation, primary microscopic examination of the samples was carried out by Cornelis Veltkamp & Carmel Pinnington at the Department of Earth, Ocean Sciences, University of Liverpool.

## 4. Conclusions 

Cellulose is the most abundant polysaccharide on Earth, the degradation of this recalcitrant substrate is therefore an important driver of the carbon cycle. Although a number of cultured bacterial species are known to degrade cellulose, there is a paucity of information on true community function, structure, particularly in the marine environment [45]. Metagenomic analysis of the biofilm that developed on cellulose immersed in the Irish Sea for one month has revealed a community dominated by members of the Gammaproteobacteria, Bacteroidetes, supported by both protein encoding, 16S rRNA gene distribution in the 454 pyrosequence dataset. Significantly, a total of 211 genes were identified as potentially involved in the polysaccharide degrading process, reflecting the metagenome of a microbial community enriched in cellulolytic microorganisms. SEM supported the metagenomic data in showing a biofilm dominated by small (<1 µm) rod-shaped bacteria that form erosion pits in the cellulose surface. There was a relatively high frequency of sequence similarity matches to genes of the marine cellulose-degrading bacterial species S. degradans, T. turnerae, the cellulolytic soil bacterium C. hutchinsonii, both of which have sequenced genomes, cellulase systems that have been well characterized recently. However it is important to note that the available sequence information in databases greatly under represents the true diversity present in environmental microbial communities, matches are to the closest relatives in databases. The metagenomic data here provide evidence for the existence of a pool of potentially exploitable polysaccharide hydrolases in the marine microbial community, revealed by an in situ approach to generating metagenomes that are enriched in genes that encode these enzymes. 

## Supplementary Files

Supplementary File 1 PDF-Document (PDF, 134 KB)
