# Supplementary material for: Identification of Carbohydrate Metabolism Genes in the Metagenome of a Marine Biofilm Community Shown to Be Dominated by Gammaproteobacteria, Bacteroidetes
Source: Genes (Basel). 2010 Oct 26;1(3):371–84. doi: 10.3390/genes1030371 (PMC3966224; doi:10.3390/genes1030371)
Supplement: Supplementary File 1 — PDF-Document (PDF, 134 KB) [file genes-01-00371-s001.pdf]

Supplementary Table 1. Summary of similarities found in the Irish Sea metagenome to Glycoside Hydrolases present in a database constructed from twelve Pfam families.

Assembled contig ID	UniProt Accession number	% identity	alignment length	mismatches	gap openings	q. start	q. end	s. start	s. end	e-value	bit score
**contig06721**	Q11NQ3_CYTH3	75.29	170	42	0	684	175	56	225	2.00E-116	335
**contig02397**	Q11UC0_CYTH3	65.79	190	65	0	291	860	97	286	1.00E-100	318
**contig05603**	Q21NA7_SACD2	54.24	177	81	0	2	532	86	262	2.00E-95	265
**contig26022**	A7LS44_BACOV	54.14	133	61	0	455	853	778	910	2.00E-79	181
**contig04690**	Q11NQ3_CYTH3	76.98	126	29	0	73	450	33	158	2.00E-67	251
**contig05277**	Q11VQ4_CYTH3	50.26	193	96	0	2	580	938	1130	1.00E-64	243
**contig03455**	Q11PI8_CYTH3	41.06	263	155	0	113	901	1050	1312	1.00E-62	187
**contig26107**	Q11VQ4_CYTH3	68.06	144	46	0	443	12	673	816	2.00E-62	234
**contig04179**	A3HX16_9SPHI	55.56	153	68	0	4	462	105	257	2.00E-62	235
**contig07415**	Q11NQ3_CYTH3	77.14	70	16	0	599	390	236	305	1.00E-60	126
**contig02925**	Q11UC0_CYTH3	80.77	104	20	0	2	313	443	546	3.00E-60	227
**contig01407**	A6EHL7_9SPHI	49.39	164	83	0	1376	885	282	445	1.00E-58	224
**contig04013**	Q11PI8_CYTH3	68.75	112	35	0	337	2	340	451	2.00E-55	211
**contig02917**	Q308B9_9BACT	68.07	119	38	0	31	387	223	341	1.00E-54	209
**contig12578**	A7V9B3_BACUN	77.19	57	13	0	217	47	777	833	2.00E-53	118
**contig05485**	Q308B7_9BACT	70.73	82	24	0	502	257	227	308	1.00E-51	115
**contig25936**	B1ZP97_OPITP	56.67	60	26	0	387	208	131	190	2.00E-50	91.8
**contig00226**	Q11PI9_CYTH3	62.65	83	31	0	366	118	139	221	1.00E-49	95.9
**contig04413**	Q21NA7_SACD2	64.76	105	37	0	389	75	556	660	6.00E-47	184
**contig26301**	Q11PI9_CYTH3	57.8	109	46	0	339	13	839	947	2.00E-45	178
**contig25564**	Q11VQ4_CYTH3	51.16	129	63	0	395	9	902	1030	3.00E-44	174
**contig08782**	Q11PI8_CYTH3	72.04	93	26	0	285	7	467	559	3.00E-42	167

Supplementary Table 1. *Cont.*

Assembled contig ID	UniProt Accession number	% identity	alignment length	mismatches	gap openings	q. start	q. end	s. start	s. end	e-value	bit score
**contig05185**	Q9REW0_ERWCH	60.34	58	23	0	200	373	184	241	5.00E-42	95.5
**contig05113**	B3PD52_CELJU	51.72	116	56	0	83	430	325	440	7.00E-41	163
**contig04687**	C5BPZ6_9GAMM	68.18	88	28	0	3	266	82	169	6.00E-40	159
**contig15223**	Q11PI8_CYTH3	67.44	86	28	0	15	272	616	701	2.00E-39	157
**contig04664**	Q023N8_SOLUE	51.72	87	42	0	262	2	467	553	5.00E-37	126
**contig08470**	Q11VF9_CYTH3	68.18	66	21	0	801	998	54	119	6.00E-37	126
**contig17088**	Q06B38_9BACT	60.22	93	37	0	3	281	324	416	1.00E-36	149
**contig01678**	Q21HS9_SACD2	53.92	102	47	0	308	3	412	513	1.00E-36	149
**contig18893**	Q47XT7_COLP3	72.15	79	22	0	239	3	227	305	7.00E-36	145
**contig08844**	A4CKZ7_9FLAO	69.74	76	23	0	236	9	166	241	1.00E-34	141
**contig24355**	Q21GC6_SACD2	67.5	80	26	0	240	1	53	132	2.00E-34	141
**contig14077**	B3PEQ9_CELJU	69.62	79	24	0	2	238	197	275	2.00E-33	137
**contig02702**	Q12KZ3_SHEDO	73.24	71	19	0	215	3	112	182	5.00E-33	136
**contig02598**	B6CH67_9BILA	49.56	113	57	0	22	360	58	170	2.00E-32	134
**contig25780**	A7LS44_BACOV	45.71	105	57	0	2	316	556	660	3.00E-32	133
**contig16934**	Q21KX4_SACD2	68.29	82	26	0	2	247	1413	1494	1.00E-31	131
**contig00765**	Q21KX4_SACD2	51.35	111	54	0	384	52	1527	1637	1.00E-31	133
**contig23140**	Q2SFD8_HAHCH	64.56	79	28	0	239	3	160	238	2.00E-31	130
**contig01065**	Q11SW6_CYTH3	55.71	70	31	0	662	453	210	279	5.00E-31	96.4
**contig00382**	A7LS44_BACOV	46.15	91	49	0	373	101	807	897	5.00E-31	114
**contig13597**	C5BNN7_9GAMM	45.54	112	61	0	456	121	332	443	8.00E-30	126
**contig06190**	Q11PI8_CYTH3	67.53	77	25	0	95	325	23	99	9.00E-30	126
**contig11004**	Q11VL4_CYTH3	64.38	73	26	0	220	2	141	213	1.00E-28	121
**contig20446**	A5ZK01_9BACE	56.96	79	34	0	3	239	204	282	4.00E-28	120
**contig22358**	C5BPZ6_9GAMM	75.86	58	14	0	65	238	53	110	5.00E-28	119

Supplementary Table 1. *Cont.*

Assembled contig ID	UniProt Accession number	% identity	alignment length	mismatches	gap openings	q. start	q. end	s. start	s. end	e-value	bit score
**contig02656**	A9LDW9_9BACT	52.58	97	46	0	12	302	485	581	9.00E-28	119
**contig10055**	Q21PQ1_SACD2	56.25	80	35	0	1	240	117	196	3.00E-27	116
**contig02758**	Q21FN3_SACD2	47.22	72	38	0	217	432	35	106	9.00E-27	80.3
**contig19798**	Q21ID4_SACD2	69.49	59	18	0	179	3	93	151	1.00E-26	114
**contig06707**	Q11UJ1_CYTH3	70	60	18	0	3	182	350	409	1.00E-26	114
**contig25876**	B8D159_HALOH	53.42	73	34	0	219	1	198	270	3.00E-26	113
**contig02648**	Q21NF6_SACD2	74.14	58	15	0	176	3	134	191	4.00E-26	112
**contig15633**	Q21F54_SACD2	52.86	70	33	0	1	210	488	557	1.00E-25	111
**contig04461**	Q11VQ4_CYTH3	59.09	44	18	0	1	132	1024	1067	1.00E-25	73.5
**contig20483**	Q084Z3_SHEFN	56.96	79	34	0	239	3	74	152	4.00E-25	110
**contig00162**	Q11SW6_CYTH3	56.76	74	32	0	5	226	492	565	1.00E-24	109
**contig04227**	B1KJF6_SHEWM	69.09	55	17	0	3	167	30	84	1.00E-24	108
**contig12437**	Q5AU13_EMENI	38.74	111	68	0	3	335	393	503	7.00E-24	97.7
**contig12453**	Q21G01_SACD2	69.09	55	17	0	165	1	260	314	7.00E-24	105
**contig26477**	A6EIU7_9SPHI	60.32	63	25	0	341	153	188	250	9.00E-24	70.7
**contig04615**	Q11VQ4_CYTH3	65.08	63	22	0	2	190	714	776	2.00E-23	104
**contig16578**	Q47XT7_COLP3	76.92	52	12	0	239	84	1867	1918	5.00E-23	103
**contig23608**	Q11VQ4_CYTH3	51.85	54	26	0	70	231	1011	1064	6.00E-23	80.8
**contig01684**	Q11X01_CYTH3	57.33	75	32	0	853	629	601	675	7.00E-23	104
**contig23338**	Q11PI8_CYTH3	64.91	57	20	0	319	149	384	440	6.00E-22	100
**contig04241**	B0RNG4_XANCB	47.06	85	45	0	324	70	536	620	6.00E-22	100
**contig17293**	B7RVI9_9GAMM	58.46	65	27	0	215	21	253	317	1.00E-21	98.7
**contig13913**	C0INS7_9BACT	43.36	113	64	0	341	3	52	164	2.00E-21	98.2
**contig03316**	Q21N31_SACD2	54.69	64	29	0	195	4	462	525	2.00E-21	97.7
**contig10093**	B3PEQ9_CELJU	52.7	74	35	0	19	240	157	230	3.00E-21	97.3

Supplementary Table 1. *Cont.*

Assembled contig ID	UniProt Accession number	% identity	alignment length	mismatches	gap openings	q. start	q. end	s. start	s. end	e-value	bit score
**contig14342**	C1S930_9SPHI	44.32	88	49	0	599	336	481	568	3.00E-21	98.7
**contig24475**	B2UQH3_AKKM8	51.56	64	31	0	18	209	214	277	5.00E-21	96.4
**contig02705**	Q2SHX0_HAHCH	73.47	49	13	0	8	154	393	441	9.00E-21	95
**contig19349**	A0Y0X8_9GAMM	55.38	65	29	0	31	225	446	510	1.00E-20	95.5
**contig01813**	Q11PI8_CYTH3	39.58	96	58	0	846	559	2672	2767	1.00E-20	96.8
**contig06502**	A9EU19_SORC5	45.57	79	43	0	299	63	115	193	3.00E-20	94.5
**contig22983**	Q21ZF8_RHOFD	59.65	57	23	0	173	3	109	165	2.00E-19	90.4
**contig10599**	A0Y0X8_9GAMM	57.38	61	26	0	184	2	446	506	6.00E-19	89.5
**contig17731**	A7LS44_BACOV	48.61	72	37	0	227	12	545	616	3.00E-18	87.2
**contig14052**	Q11T98_CYTH3	62.96	27	10	0	250	330	93	119	4.00E-18	46.4
**contig11080**	C1S930_9SPHI	46.75	77	41	0	231	1	486	562	6.00E-18	86.3
**contig00230**	O66185_BACCI	57.41	54	23	0	10	171	275	328	8.00E-18	85.4
**contig01917**	Q21HS5_SACD2	50.82	61	30	0	192	10	348	408	4.00E-17	83.1
**contig05872**	Q11UJ1_CYTH3	74.42	43	11	0	131	3	352	394	4.00E-17	82.6
**contig07156**	Q11VQ4_CYTH3	45.95	74	40	0	783	562	1425	1498	5.00E-17	84.9
**contig25655**	Q8GRB4_9PSED	45	80	44	0	575	336	1	80	7.00E-17	84
**contig05967**	C0INS7_9BACT	66.2	71	24	0	222	10	500	570	7.00E-17	82.6
**contig18210**	Q21F54_SACD2	60.78	51	20	0	5	157	514	564	9.00E-17	81.7
**contig01984**	Q21KX4_SACD2	38.38	99	61	0	312	16	1512	1610	1.00E-16	82.6
**contig24905**	Q21KX4_SACD2	55.81	43	19	0	132	4	1661	1703	2.00E-16	63.8
**contig05199**	Q9BH59_MELIC	55.07	69	31	0	3	209	59	127	2.00E-16	80.8
**contig06009**	Q11PI9_CYTH3	43.66	71	40	0	396	184	2683	2753	6.00E-16	80.3
**contig09168**	Q11NQ3_CYTH3	49.15	59	30	0	191	15	636	694	7.00E-16	79
**contig15044**	Q2SFD8_HAHCH	60	50	20	0	169	20	437	486	8.00E-16	79
**contig01223**	Q11NQ3_CYTH3	63.64	44	16	0	96	227	571	614	9.00E-16	79

Supplementary Table 1. *Cont.*

Assembled contig ID	UniProt Accession number	% identity	alignment length	mismatches	gap openings	q. start	q. end	s. start	s. end	e-value	bit score
**contig04098**	Q308B9_9BACT	59.26	54	22	0	299	138	293	346	1.00E-15	79
**contig04503**	Q9X3P6_9FIRM	70.37	27	8	0	1	81	111	137	1.00E-15	53.3
**contig06091**	C4EIC3_STRRS	43.55	62	35	0	735	920	63	124	1.00E-15	80.8
**contig00650**	A9B2K3_HERA2	49.21	63	32	0	19	207	55	117	1.00E-15	78.5
**contig05391**	A7V9B3_BACUN	56	50	22	0	292	143	253	302	2.00E-15	78.5
**contig14108**	A4CA09_9GAMM	75	40	10	0	4	123	304	343	3.00E-15	77.1
**contig11183**	Q97G04_CLOAB	52.83	53	25	0	198	40	35	87	4.00E-15	78.5
**contig14858**	Q11PI8_CYTH3	87.88	33	4	0	95	193	936	968	7.00E-15	75.8
**contig13599**	B3C9G1_9BACE	43.55	62	35	0	1	186	450	511	9.00E-15	75.8
**contig09264**	B6EH15_ALISL	50	62	31	0	231	46	362	423	1.00E-14	75.3
**contig07887**	Q21KE5_SACD2	72.22	36	10	0	1	108	326	361	2.00E-14	74.4
**contig01054**	Q11PI8_CYTH3	50	58	29	0	636	809	2702	2759	3.00E-14	76.7
**contig01703**	Q21KX4_SACD2	86.67	30	4	0	129	40	1088	1117	3.00E-14	68.4
**contig07593**	B0ZSE6_9ACTO	75	32	8	0	97	2	492	523	3.00E-14	73.5
**contig25464**	A6MI38_9SPIT	45.9	61	33	0	201	19	167	227	5.00E-14	73
**contig14490**	Q18BV5_CLOD6	40.26	77	46	0	232	2	62	138	6.00E-14	73
**contig07103**	Q21HS9_SACD2	42.31	78	45	0	156	389	235	312	9.00E-14	73
**contig10391**	B6VYB2_9BACE	48.98	49	25	0	180	34	94	142	1.00E-13	72.1
**contig10691**	Q11UJ1_CYTH3	49.18	61	31	0	654	472	716	776	1.00E-13	73.5
**contig02059**	O24820_9BACT	53.57	56	26	0	341	508	5	60	1.00E-13	73
**contig04779**	Q11PI9_CYTH3	47.37	57	30	0	507	337	1355	1411	1.00E-13	73.5
**contig11483**	Q3R055_XYLFA	58.54	41	17	0	51	173	280	320	2.00E-13	71.2
**contig01895**	A4F259_9BACL	55.32	47	21	0	2	142	334	380	3.00E-13	70.3
**contig09849**	B6QSI9_PENMQ	36.89	103	65	0	601	293	241	343	5.00E-13	71.2
**contig23872**	B8D1V5_HALOH	43.9	41	23	0	123	1	870	910	6.00E-13	53.8

Supplementary Table 1. *Cont.*

Assembled contig ID	UniProt Accession number	% identity	alignment length	mismatches	gap openings	q. start	q. end	s. start	s. end	e-value	bit score
**contig03397**	Q11PI9_CYTH3	35.71	84	54	0	614	363	2669	2752	6.00E-13	71.2
**contig16794**	C5BPZ6_9GAMM	65.71	35	12	0	105	1	189	223	8.00E-13	68.9
**contig18313**	B9MKT7_ANATD	50.98	51	25	0	8	160	102	152	9.00E-13	68.4
**contig14908**	C1Z0L6_PEDHE	62.79	43	16	0	215	343	6	48	1.00E-12	69.3
**contig17865**	Q11VF9_CYTH3	67.65	34	11	0	104	3	409	442	1.00E-12	62.5
**contig08385**	Q11PI9_CYTH3	52.17	69	33	0	209	3	580	648	2.00E-12	68.4
**contig10324**	Q5ZGC9_9CAUD	65.71	35	12	0	106	2	115	149	5.00E-12	66.1
**contig16511**	Q4UVY1_XANC8	50	38	19	0	171	58	79	116	5.00E-12	48.3
**contig04307**	A7V9A3_BACUN	73.08	26	7	0	2	79	537	562	7.00E-12	47.3
**contig01403**	B5JIL5_9BACT	54.55	44	20	0	2	133	321	364	8.00E-12	65.7
**contig08855**	B9K9B3_THENN	36	75	48	0	62	286	200	274	9.00E-12	66.1
**contig13936**	A9FK25_SORC5	52.27	44	21	0	2	133	18	61	1.00E-11	64.8
**contig07270**	C1UVR8_9DELT	67.65	34	11	0	6	107	46	79	1.00E-11	63.8
**contig20317**	Q092X1_STIAU	32.39	71	48	0	220	8	251	321	2.00E-11	64.3
**contig13837**	B8CL90_SHEPW	63.64	33	12	0	100	2	43	75	4.00E-11	63.4
**contig08899**	C1WMF5_9ACTO	47.17	53	28	0	224	382	67	119	4.00E-11	64.3
**contig12904**	A9EY77_9RHOB	56.1	41	18	0	124	2	275	315	5.00E-11	63.4
**contig05947**	Q2H8K5_CHAGB	27.48	131	95	0	1895	2287	558	688	5.00E-11	66.6
**contig02283**	Q9APG3_9PSED	45.28	53	29	0	6	164	376	428	6.00E-11	62.9
**contig09275**	Q47XT7_COLP3	47.06	51	27	0	160	8	160	210	6.00E-11	62.9
**contig12768**	Q11PI8_CYTH3	36.67	60	38	0	222	401	2693	2752	1.00E-10	62.9
**contig15585**	A5WEY2_PSYWF	72.73	33	9	0	162	64	82	114	1.00E-10	62
**contig00744**	A0Y0X8_9GAMM	78.57	28	6	0	3	86	683	710	2.00E-10	61.1
**contig26451**	B9XAL6_9BACT	43.14	51	29	0	154	2	552	602	3.00E-10	60.2
**contig26454**	C1W4K3_9MICO	52.94	34	16	0	28	129	316	349	3.00E-10	48.3

Supplementary Table 1. *Cont.*

Assembled contig ID	UniProt Accession number	% identity	alignment length	mismatches	gap openings	q. start	q. end	s. start	s. end	e-value	bit score
**contig16506**	GUN2_CLOJO	52.94	34	16	0	21	122	201	234	4.00E-10	54.2
**contig20063**	Q12KZ3_SHEDO	53.19	47	22	0	2	142	309	355	5.00E-10	59.7
**contig10727**	B1VW82_STRGG	53.85	39	18	0	1	117	72	110	6.00E-10	58.8
**contig04801**	Q21F54_SACD2	65.63	32	11	0	2	97	535	566	6.00E-10	58.8
**contig14405**	C5FQP0_MICCA	30.56	72	50	0	269	54	595	666	7.00E-10	59.7
**contig11559**	Q2SFD8_HAHCH	58.33	36	15	0	148	41	441	476	3.00E-09	57.4
**contig25649**	Q21KX4_SACD2	67.65	34	11	0	2	103	1448	1481	4.00E-09	56.5
**contig09617**	B7AFY4_9BACE	41.18	51	30	0	342	190	46	96	4.00E-09	58.3
**contig15196**	Q21KX4_SACD2	66.67	30	10	0	98	9	848	877	7.00E-09	56.1
**contig09479**	A5FL64_FLAJ1	30.67	75	52	0	16	240	378	452	7.00E-09	56.1
**contig25208**	Q11PI8_CYTH3	46.94	49	26	0	53	199	2682	2730	8.00E-09	55.6
**contig05463**	Q2SCY8_HAHCH	66.67	30	10	0	90	1	185	214	1.00E-08	55.6
**contig14537**	Q9X3P6_9FIRM	62.5	32	12	0	232	137	212	243	2.00E-08	54.7
**contig25770**	Q11PI9_CYTH3	56.41	39	17	0	118	2	8	46	2.00E-08	56.1
**contig03641**	B9AN63_9BACT	33.93	56	37	0	313	146	249	304	2.00E-08	56.1
**contig19790**	C5BTS3_9GAMM	51.22	41	20	0	124	2	277	317	3.00E-08	53.3
**contig21717**	A4BCU4_9GAMM	85.71	21	3	0	64	2	268	288	3.00E-08	52.8
**contig09391**	B7RVI9_9GAMM	55.88	34	15	0	10	111	264	297	3.00E-08	54.2
**contig25883**	Q47XT7_COLP3	64.71	34	12	0	110	9	160	193	4.00E-08	54.2
**contig26116**	Q11PI9_CYTH3	35.71	56	36	0	579	412	2686	2741	4.00E-08	56.5
**contig12854**	Q2SHX0_HAHCH	51.43	35	17	0	179	283	758	792	5.00E-08	54.2
**contig15246**	Q12KZ3_SHEDO	33.33	75	50	0	234	10	565	639	5.00E-08	53.3
**contig13222**	B7AFY4_9BACE	76.92	26	6	0	227	150	241	266	6.00E-08	52.8
**contig16115**	C1S6C0_9SPHI	42	50	29	0	31	180	503	552	6.00E-08	52.8
**contig04127**	Q21N15_SACD2	52.94	34	16	0	88	189	199	232	9.00E-08	52.4

Supplementary Table 1. *Cont.*

Assembled contig ID	UniProt Accession number	% identity	alignment length	mismatches	gap openings	q. start	q. end	s. start	s. end	e-value	bit score
**contig14791**	C0T375_9PSEU	43.48	46	26	0	216	79	32	77	1.00E-07	51.9
**contig20202**	Q5BDV7_EMENI	66.67	30	10	0	117	206	145	174	1.00E-07	51.9
**contig01911**	Q841J5_9ALTE	67.86	28	9	0	200	283	362	389	1.00E-07	52.4
**contig22348**	B7RVI9_9GAMM	46.81	47	25	0	142	2	1099	1145	1.00E-07	51.5
**contig25525**	Q21GU0_SACD2	39.71	68	41	0	259	56	38	105	1.00E-07	52.8
**contig07601**	Q308B8_9BACT	41.3	46	27	0	138	1	43	88	1.00E-07	51.9
**contig16702**	Q5ART1_EMENI	31.11	90	62	0	156	425	95	184	3.00E-07	51.9
**contig18707**	A5VF24_SPHWW	74.07	27	7	0	60	140	491	517	3.00E-07	50.1
**contig18663**	B9KKR0_RHOSK	56.45	62	27	0	5	190	352	413	4.00E-07	50.1
**contig25920**	Q2SJA2_HAHCH	51.43	35	17	0	134	30	717	751	4.00E-07	50.1
**contig22036**	O88021_STRCO	50	34	17	0	183	82	416	449	5.00E-07	49.6
**contig23021**	Q21GJ3_SACD2	51.52	33	16	0	3	101	84	116	5.00E-07	48.7
**contig04598**	Q11PI8_CYTH3	37.29	59	37	0	249	73	2717	2775	5.00E-07	52.4
**contig10181**	A2TX55_9FLAO	36.76	68	43	0	158	361	815	882	7.00E-07	50.1
**contig11249**	Q21GB9_SACD2	50	34	17	0	5	106	706	739	7.00E-07	48.3
**contig07930**	B5JIL5_9BACT	51.43	35	17	0	2	106	51	85	7.00E-07	50.1
**contig20761**	O69347_VIBSM	71.43	21	6	0	79	141	366	386	8.00E-07	48.7
**contig25459**	Q0FSH6_9RHOB	52.78	36	17	0	121	228	858	893	8.00E-07	49.2
**contig20414**	A7LS44_BACOV	50.82	61	30	0	1	183	927	987	9.00E-07	48.7
**contig15701**	Q21KX4_SACD2	56	25	11	0	128	54	711	735	1.00E-06	37.7
**contig25008**	A8XBB0_CAEBR	50	44	22	0	146	15	542	585	1.00E-06	48.7
**contig00570**	Q11PI8_CYTH3	45.95	37	20	0	330	220	2724	2760	1.00E-06	49.2
**contig15304**	A9FX20_SORC5	64.52	31	11	0	2	94	204	234	2.00E-06	47.3
**contig21246**	B1ZLP5_METPB	44.12	68	38	0	212	9	329	396	2.00E-06	47.8
**contig23584**	Q245S3_TETTH	45	40	22	0	124	5	1680	1719	2.00E-06	47.3

Supplementary Table 1. *Cont.*

Assembled contig ID	UniProt Accession number	% identity	alignment length	mismatches	gap openings	q. start	q. end	s. start	s. end	e-value	bit score
**contig00357**	Q3R055_XYLFA	55.56	27	12	0	19	99	188	214	2.00E-06	46.9
**contig08179**	C1UVR8_9DELT	63.33	30	11	0	188	277	31	60	2.00E-06	49.2
**contig15332**	Q2SCY8_HAHCH	71.43	21	6	0	196	134	264	284	3.00E-06	46.9
**contig16110**	B0CET5_ACAM1	69.23	26	8	0	173	96	629	654	3.00E-06	46.9
**contig16669**	Q21KX4_SACD2	69.23	26	8	0	27	104	1274	1299	3.00E-06	46.4
**contig17172**	A1ZFU5_9SPHI	38.6	57	35	0	173	3	229	285	3.00E-06	47.3
**contig06033**	Q55365_SYNY3	68	25	8	0	521	447	1017	1041	4.00E-06	48.3
**contig04332**	A9B399_HERA2	51.52	33	16	0	103	5	226	258	5.00E-06	45.5
**contig01518**	A7LS44_BACOV	37.7	61	38	0	65	247	810	870	6.00E-06	46.4
**contig11361**	Q21GB9_SACD2	40.82	49	29	0	198	52	677	725	7.00E-06	46
**contig25454**	B5HIM9_STRPR	48.72	39	20	0	40	156	19	57	7.00E-06	47.3
**contig15581**	Q245S3_TETTH	50	32	16	0	3	98	1683	1714	8.00E-06	45.1
**contig02861**	A7LS44_BACOV	64.29	28	10	0	131	48	683	710	1.00E-05	45.5
**contig03003**	A0Y0X8_9GAMM	84.21	19	3	0	3	59	691	709	1.00E-05	44.6

Supplementary Table 2. Summary of 211 sequences with Glycoside Hydrolase similarity used as a blast query against the NCBI-nr database.

Assembled contig ID	NCBI Accession number and Organism	% identity	alignment length	q. start	q. end	s. start	s. end	e-value	bit score
**contig03003**	gi|88859385 Pseudoalteromonas tunicata	71.43	21	3	65	858	878	0.038	41.2
**contig06721**	gi|110640093 Cytophaga hutchinsonii	70.94	234	684	1	56	289	4.00E-98	361
**contig02397**	gi|110638127 Cytophaga hutchinsonii	66	250	117	860	38	286	7.00E-101	354
**contig05603**	gb ABD79822.1 Saccharophagus degradans 2 40	54.24	177	2	532	86	262	1.00E-85	225
**contig26022**	gi|225012305 Flavobacteria bacterium MS024 2A	53.56	267	476	1273	486	752	3.00E-124	304
**contig04690**	gi|110640093 Cytophaga hutchinsonii	68.92	148	10	450	11	158	9.00E-55	216
**contig05277**	gi|86140693 Leeuwenhoekiella blandensis	73.97	242	918	1643	2	243	5.00E-104	382
**contig03455**	Cytophaga hutchinsonii	54.4	307	2	901	1016	1312	2.00E-73	280
**contig26107**	gi|110637645 Cytophaga hutchinsonii	68.06	144	443	12	673	816	9.00E-50	199
**contig04179**	gi|126646843 Algoriphagus sp PR1	55.56	153	4	462	105	257	1.00E-49	199
**contig07415**	gi|110640093 Cytophaga hutchinsonii	71.03	107	599	279	236	340	6.00E-56	153
**contig02925**	gi|110638127 Cytophaga hutchinsonii	80.77	104	2	313	443	546	6.00E-48	193
**contig01407**	gi|227539966 Sphingobacterium spiritivorum	64.6	161	1370	888	284	444	2.00E-59	234
**contig04013**	gi|110639808 Cytophaga hutchinsonii	68.75	112	337	2	340	451	7.00E-44	179
**contig02917**	gi|78926855 uncultured bacterium	68.07	119	31	387	223	341	3.00E-43	177
**contig12578**	gi|254788171 Teredinibacter turnerae T7901	54.55	99	555	259	629	727	1.00E-57	120
**contig05485**	gi|78926927 uncultured bacterium	60.24	166	502	8	227	392	6.00E-44	180
**contig25936**	gi|242260148 Clostridium cellulovorans 743B	52.51	179	588	52	85	250	8.00E-46	187
**contig00226**	gi|110639807 Cytophaga hutchinsonii	67.11	152	459	10	107	258	3.00E-44	181
**contig04413**	gi|124006662 Microscilla marina	73.88	134	751	350	10	143	2.00E-50	202
**contig26301**	gi|110639807 Cytophaga hutchinsonii	60.36	111	339	7	839	947	3.00E-36	154
**contig25564**	gi|110638770 Cytophaga hutchinsonii	59.69	129	395	9	314	442	2.00E-40	168
**contig08782**	gi|110639808 Cytophaga hutchinsonii	72.04	93	285	7	467	559	1.00E-32	142
**contig05185**	gi|242239319 Dickeya dadantii	56.91	123	8	373	119	241	6.00E-37	156
**contig05113**	gi|254787279 Teredinibacter turnerae T7901	57.14	133	74	451	501	633	2.00E-39	164

Supplementary Table 2. *Cont.*

Assembled contig ID	NCBI Accession number and Organism	% identity	alignment length	q. start	q. end	s. start	s. end	e-value	bit score
**contig04687**	gi|254787186 protein Teredinibacter turnerae	68.18	88	3	266	82	169	9.00E-31	136
**contig15223**	gi|110639808 Cytophaga hutchinsonii	67.44	86	15	272	616	701	3.00E-30	134
**contig04664**	gi|90019972 Saccharophagus degradans 2 40	64.39	132	397	2	498	629	1.00E-48	195
**contig08470**	gi|110637740 Cytophaga hutchinsonii	54.4	125	627	998	1	119	1.00E-31	141
**contig17088**	gi|115334997 uncultured bacterium	46.99	166	3	491	324	486	2.00E-40	168
**contig01678**	gi|90022135 Saccharophagus degradans 2 40	53.92	102	308	3	412	513	4.00E-28	127
**contig18893**	gi|71281571 Colwellia psychrerythraea 34H	72.15	79	239	3	227	305	3.00E-27	124
**contig08844**	gi|260062808 Robiginitalea biformata	69.74	76	236	9	166	241	3.00E-26	121
**contig24355**	gi|90022638 Saccharophagus degradans	67.5	80	240	1	53	132	4.00E-26	120
**contig14077**	gi|192361876 Cellvibrio japonicus	69.62	79	2	238	197	275	3.00E-25	117
**contig02702**	gi|91793955 Shewanella denitrificans	73.24	71	215	3	112	182	7.00E-25	116
**contig02598**	gi|242239319 Dickeya dadantii	51.61	124	1	372	70	193	2.00E-31	138
**contig25780**	gi|255607952 Ricinus communis	66.67	105	2	316	210	314	2.00E-33	145
**contig16934**	gi|90021040 Saccharophagus degradans 2 40	68.29	82	2	247	1413	1494	1.00E-23	112
**contig00765**	gi|88803060 Polaribacter irgensii	61.63	172	510	1	262	433	9.00E-56	219
**contig23140**	gi|83646626 Hahella chejuensis	64.56	79	239	3	160	238	2.00E-23	112
**contig01065**	gi|110638631 Cytophaga hutchinsonii	45.32	267	773	6	177	438	6.00E-57	224
**contig00382**	gi|86143269 Leeuwenhoekiella blandensis	68.99	129	400	14	620	748	3.00E-48	194
**contig13597**	gi|254784817 Teredinibacter turnerae T7901	45.54	112	456	121	332	443	1.00E-26	108
**contig06190**	gi|110639808 Cytophaga hutchinsonii	70	80	95	325	20	99	2.00E-22	108
**contig11004**	gi|110637685 Cytophaga hutchinsonii	64.38	73	220	2	141	213	4.00E-21	104
**contig20446**	gi|237719706 Bacteroides sp	56.96	79	3	239	201	279	8.00E-21	103
**contig22358**	gi|254787186 Teredinibacter turnerae T7901	75.86	58	65	238	53	110	6.00E-25	102
**contig02656**	gi|78926900 uncultured bacterium	53.06	98	9	302	469	559	4.00E-23	110
**contig10055**	gi|90019713 Saccharophagus degradans 2 40	56.25	80	1	240	117	196	5.00E-20	100
**contig02758**	gi|90022881 Saccharophagus degradans 2 40	47.22	72	217	432	35	106	2.00E-19	69.7

Supplementary Table 2. *Cont.*

Assembled contig ID	NCBI Accession number and Organism	% identity	alignment length	q. start	q. end	s. start	s. end	e-value	bit score
**contig19798**	gi|90021930 Saccharophagus degradans 2 40	69.49	59	179	3	93	151	2.00E-19	98.6
**contig06707**	gi|110638056 Cytophaga hutchinsonii	70	60	3	182	350	409	2.00E-19	98.6
**contig25876**	gi|242259850 Clostridium cellulovorans	57.5	80	222	1	249	327	5.00E-20	100
**contig02648**	gi|90020158 Saccharophagus degradans 2 40	74.14	58	176	3	134	191	6.00E-19	97.1
**contig15633**	gi|90023060 Saccharophagus degradans 2 40	52.86	70	1	210	488	557	1.00E-18	96.3
**contig04461**	gi|110638770 Cytophaga hutchinsonii	45.53	123	1	363	436	555	2.00E-21	106
**contig20483**	gi|212634478 Shewanella piezotolerans	56.96	79	239	3	69	147	3.00E-18	94.7
**contig00162**	gi|110638631 Cytophaga hutchinsonii	56.76	74	5	226	492	565	5.00E-18	94
**contig04227**	gi|170728697 Shewanella woodyi	69.09	55	3	167	30	84	9.00E-18	93.2
**contig12437**	gi|110637223 Cytophaga hutchinsonii	76.32	114	3	344	264	377	4.00E-88	200
**contig12453**	gi|90022763 Saccharophagus degradans 2 40	69.09	55	165	1	260	314	4.00E-17	90.9
**contig26477**	gi|261404702 Geobacillus sp	65.26	95	437	153	161	251	7.00E-30	111
**contig04615**	gi|110637645 Cytophaga hutchinsonii	65.08	63	2	190	714	776	1.00E-16	89.7
**contig16578**	gi|71281571 Colwellia psychrerythraea	76.92	52	239	84	1867	1918	2.00E-16	89
**contig23608**	gi|110638770 Cytophaga hutchinsonii	57.69	78	1	231	399	476	2.00E-21	105
**contig01684**	gi|110637198 Cytophaga hutchinsonii	44.59	148	886	452	592	736	1.00E-26	124
**contig23338**	gi|110639808 Cytophaga hutchinsonii	46.02	113	319	2	384	496	3.00E-19	98.2
**contig04241**	gi|159897669 Herpetosiphon aurantiacus	50.93	108	318	1	111	218	3.00E-24	114
**contig17293**	gi|83645744 Hahella chejuensis	60	65	215	21	576	640	3.00E-16	88.2
**contig13913**	gi|192361876 Cellvibrio japonicus	45.13	113	341	3	85	197	2.00E-20	102
**contig03316**	gi|90020283 Saccharophagus degradans 2 40	54.69	64	195	4	462	525	4.00E-15	84.3
**contig10093**	gi|192361876 Cellvibrio japonicus	52.7	74	19	240	157	230	5.00E-15	84
**contig14342**	gi|110637461 Cytophaga hutchinsonii	46.15	91	599	330	868	958	6.00E-19	97.8
**contig24475**	gi|153808937 Bacteroides caccae	50.68	73	21	239	201	273	5.00E-16	87.4
**contig02705**	gi|83645744 Hahella chejuensis	73.47	49	8	154	393	441	2.00E-14	82
**contig19349**	gi|119469861 Alteromonadales bacterium	53.95	76	1	225	435	510	2.00E-16	88.6

Supplementary Table 2. *Cont.*

Assembled contig ID	NCBI Accession number and Organism	% identity	alignment length	q. start	q. end	s. start	s. end	e-value	bit score
**contig01813**	gi|110639806 Cytophaga hutchinsonii	39.17	120	846	505	2319	2437	8.00E-19	98.6
**contig06502**	gi|254787279 Teredinibacter turnerae	49.54	109	323	3	311	414	5.00E-26	120
**contig22983**	gi|90022638 Saccharophagus degradans 2 40	59.65	57	173	3	102	158	1.00E-14	82.8
**contig10599**	gi|119469861 Alteromonadales bacterium	55.13	78	232	2	429	506	2.00E-15	85.1
**contig17731**	gi|110640149 Cytophaga hutchinsonii	56	75	227	3	231	305	4.00E-16	87.8
**contig14052**	gi|162449739 Sorangium cellulosum	54.24	59	154	330	151	208	1.00E-16	68.9
**contig11080**	gi|110637461 Cytophaga hutchinsonii	43.59	78	231	1	873	950	2.00E-13	79
**contig00230**	gi|3090434 Bacillus circulans	57.14	56	10	174	275	330	4.00E-12	74.3
**contig01917**	gi|90022139 Saccharophagus degradans 2 40	50.82	61	192	10	348	408	2.00E-11	72
**contig05872**	gi|110638056 Cytophaga hutchinsonii	74.42	43	131	3	352	394	3.00E-11	71.6
**contig07156**	gi|256424202 Chitinophaga pinensis	42.67	225	783	121	19	243	2.00E-37	160
**contig25655**	gi|90019761Saccharophagus degradans 2 40	51.22	82	334	89	696	776	3.00E-27	79.7
**contig05967**	gi|56675038 uncultured bacterium	70.42	71	222	10	497	564	9.00E-12	73.2
**contig18210**	gi|90023060 Saccharophagus degradans 2 40	60.78	51	5	157	514	564	5.00E-11	70.9
**contig01984**	gi|254785013 Teredinibacter turnerae T7901	58.74	143	432	4	279	420	8.00E-40	166
**contig24905**	gi|91792085 Shewanella denitrificans OS217	63.49	63	192	4	483	544	3.00E-16	88.2
**contig05199**	gi|17826951 Pseudomonas sp ND137	61.43	70	3	209	181	250	5.00E-12	73.9
**contig06009**	gi|110639300 Cytophaga hutchinsonii	43.3	97	411	133	467	561	2.00E-18	95.5
**contig09168**	gi|110638770 Cytophaga hutchinsonii	55.74	61	191	9	453	513	1.00E-12	75.9
**contig15044**	gi|83646626 Hahella chejuensis	60	50	169	20	437	486	2.00E-10	68.6
**contig01223**	gi|110638770 Cytophaga hutchinsonii	59.15	71	6	218	355	425	2.00E-19	99
**contig04098**	gi|78926855 uncultured bacterium	52.94	102	299	3	293	394	2.00E-22	108
**contig04503**	gi|254443020 Verrucomicrobiae bacterium	72.06	68	1	201	92	159	5.00E-24	114
**contig06091**	gi|68525474 Flavobacterium sp MSY2	67.63	173	1	519	199	371	1.00E-65	254
**contig00650**	gi|256393254 protein Catenulispora acidiphila	56.92	65	19	213	705	769	1.00E-13	79.3
**contig05391**	gi|254788171 protein Teredinibacter turnerae	55	80	289	53	225	304	7.00E-18	93.6

Supplementary Table 2. *Cont.*

Assembled contig ID	NCBI Accession number and Organism	% identity	alignment length	q. start	q. end	s. start	s. end	e-value	bit score
**contig14108**	gi|8885938 Pseudoalteromonas tunicata	64.71	51	4	156	304	349	3.00E-10	68.2
**contig11183**	gi|125973914 Clostridium thermocellum	45.56	90	255	1	11	100	2.00E-13	80.5
**contig14858**	gi|110639808 Cytophaga hutchinsonii	74.24	66	2	193	903	968	4.00E-21	104
**contig13599**	gi|189464233 Bacteroides intestinalis	63.29	79	1	237	77	155	6.00E-24	113
**contig09264**	gi|110637461 Cytophaga hutchinsonii	48.72	78	231	1	732	809	1.00E-12	75.9
**contig07887**	gi|90021219 Saccharophagus degradans 2 40	72.22	36	1	108	326	361	3.00E-09	64.7
**contig01054**	gi|110638938 2552 Cytophaga hutchinsonii	28.72	282	66	887	476	732	4.00E-20	103
**contig01703**	gi|88799706 oxidoreductase Reinekea sp	90	30	129	40	455	484	2.00E-09	60.8
**contig07593**	gi|166835917 Streptomyces sp s6 204	75	32	97	2	492	523	5.00E-09	63.9
**contig25464**	gi|254787942 Teredinibacter turnerae	68.66	67	204	4	182	248	2.00E-19	98.6
**contig14490**	gi|110639833 Cytophaga hutchinsonii	83.33	78	235	2	57	134	2.00E-30	135
**contig07103**	gi|90022135 Saccharophagus degradans 2 40	39.26	135	3	389	179	312	2.00E-16	89
**contig10391**	gi|254786327 Teredinibacter turnerae T7901	74.68	79	240	4	352	426	2.00E-27	125
**contig10691**	gi|110638056 Cytophaga hutchinsonii	35.38	130	654	277	716	845	2.00E-12	76.6
**contig02059**	gi|237718505 Bacteroides sp	46.73	107	212	520	13	119	1.00E-21	106
**contig04779**	gi|110639807 Cytophaga hutchinsonii	37.6	125	609	241	1324	1445	3.00E-15	85.9
**contig11483**	gi|15837868|ref|NP_298556.1|	58.54	41	51	173	280	320	2.00E-08	62
**contig01895**	gi|283846038 Bacillus cellulosilyticus	56	50	2	151	330	379	9.00E-09	63.2
**contig09849**	gi|124003516 dehydrogenase Microscilla marina	59.15	213	622	2	239	451	1.00E-63	246
**contig23872**	gi|163786454 Flavobacteriales bacterium	84.48	58	174	1	160	217	4.00E-23	110
**contig03397**	gi|149277963 Pedobacter sp BAL39	41.51	106	677	363	1072	1176	4.00E-14	82.4
**contig16794**	gi|254787186 Teredinibacter turnerae T7901	65.71	35	105	1	189	223	8.00E-08	60.1
**contig18313**	gi|254445795 Verrucomicrobiae bacterium	62.75	51	5	157	66	116	8.00E-13	76.6
**contig14908**	gi|227539966 Sphingobacterium spiritivorum	71.11	45	209	343	5	49	3.00E-12	74.7
**contig17865**	gi|110637740 Cytophaga hutchinsonii	55.56	81	227	3	362	442	5.00E-16	87.4
**contig08385**	gi|110639807 Cytophaga hutchinsonii	46.92	130	380	3	519	648	3.00E-23	111

Supplementary Table 2. *Cont.*

Assembled contig ID	NCBI Accession number and Organism	% identity	alignment length	q. start	q. end	s. start	s. end	e-value	bit score
**contig10324**	gi|53793624 Flavobacterium phage.11b	65.71	35	106	2	115	149	4.00E-07	57.8
**contig16511**	gi|21231824 Xanthomonas campestris	50	38	171	58	79	116	4.00E-07	42.7
**contig04307**	gi|254788171 Teredinibacter turnerae T7901	87.5	24	8	79	512	535	3.00E-07	43.9
**contig01403**	gi|254788056 Teredinibacter turnerae T7901	59.38	64	5	196	339	402	3.00E-15	84.7
**contig08855**	gi|254786327 Teredinibacter turnerae T7901	74.42	86	56	313	494	579	5.00E-31	137
**contig13936**	gi|162449739 Sorangium cellulosum	59.09	44	2	133	134	175	2.00E-07	58.5
**contig07270**	gi|262197465 Haliangium ochraceum	67.65	34	6	107	67	100	2.00E-06	55.8
**contig20317**	gi|83643752 Hahella chejuensis KCTC 2396	40.79	76	229	2	401	472	7.00E-10	67
**contig13837**	gi|212634478 Shewanella piezotolerans WP3	63.64	33	100	2	43	75	2.00E-06	55.5
**contig08899**	gi|256421052 Chitinophaga pinensis DSM 2588	47.54	61	206	382	29	89	9.00E-07	56.6
**contig12904**	gi|269218354 Actinomyces sp	46.84	79	238	2	234	310	7.00E-12	73.6
**contig05947**	gi|126648235 Algoriphagus sp	72.54	244	2	733	272	514	8.00E-91	339
**contig02283**	gi|261416250 Fibrobacter succinogenes	39.22	51	6	158	428	478	4.00E-08	45.1
**contig09275**	gi|119502900 marine gamma proteobacterium	53.95	76	229	2	390	465	2.00E-11	72
**contig12768**	gi|110639806 Cytophaga hutchinsonii	36.67	60	222	401	2340	2399	3.00E-06	55.1
**contig15585**	gi|148653080 Psychrobacter sp	72.73	33	162	64	82	114	4.00E-06	54.3
**contig00744**	gi|88859385 Pseudoalteromonas tunicata	72.73	33	3	98	680	712	2.00E-06	55.5
**contig26451**	gi|223934655 bacterium Ellin514	43.14	51	154	2	552	602	1.00E-05	52.8
**contig26454**	gi|282869827 Streptomyces sp	43.04	79	25	207	295	373	3.00E-08	61.6
**contig16506**	gi|585231 Clostridium josui	52.94	34	21	122	201	234	2.00E-05	47.8
**contig20063**	gi|192360365 Cellvibrio japonicus	45.45	55	2	160	376	430	2.00E-05	52.4
**contig10727**	gi|260913278 Pasteurella dagmatis	72.97	37	4	114	18	54	4.00E-08	61.2
**contig04801**	gi|90023060 Saccharophagus degradans 2 40	65.63	32	2	97	535	566	3.00E-05	51.6
**contig14405**	gi|202953357 Debaryomyces hansenii	32.95	88	269	9	229	316	2.00E-10	68.6
**contig11559**	gi|253573954 Paenibacillus sp	50.75	67	226	41	231	297	5.00E-07	57.4
**contig25649**	gi|90021040 Saccharophagus degradans 2 40	67.65	34	2	103	1448	1481	5.00E-09	49.7

Supplementary Table 2. *Cont.*

Assembled contig ID	NCBI Accession number and Organism	% identity	alignment length	q. start	q. end	s. start	s. end	e-value	bit score
**contig09617**	gi|110637643 Cytophaga hutchinsonii	65.41	133	390	1	27	159	8.00E-45	183
**contig15196**	gi|90021040 Saccharophagus degradans 2 40	49.33	75	230	9	968	1032	1.00E-10	69.7
**contig09479**	gi|163753699 Kordia algicida	51.25	80	1	240	254	333	9.00E-18	93.2
**contig25208**	gi|110639806 Cytophaga hutchinsonii	46.94	49	53	199	2329	2377	2.00E-04	48.9
**contig05463**	gi|83645744 Hahella chejuensis	51.47	68	195	4	701	768	2.00E-12	75.1
**contig14537**	gi|150002649 Bacteroides vulgatus	48.08	52	156	1	219	270	5.00E-15	57.4
**contig25770**	gi|124002135 Microscilla marina	65.47	139	363	776	1	137	3.00E-44	182
**contig03641**	gi|254785542 Teredinibacter turnerae T7901	72.79	136	544	137	204	339	5.00E-53	210
**contig19790**	gi|188990414 Xanthomonas campestris	47.37	57	166	2	82	138	2.00E-06	55.5
**contig21717**	gi|88798396 glucanase Reinekea sp	85.71	21	64	2	268	288	0.001	46.6
**contig09391**	gi|110637461 Cytophaga hutchinsonii	48.61	72	13	228	844	915	4.00E-16	87.8
**contig25883**	gi|119502900 marine gamma proteobacterium	45.07	71	221	9	576	644	3.00E-12	61.6
**contig26116**	gi|124009083 Microscilla marina	33.12	157	780	334	1498	1648	2.00E-17	95.1
**contig12854**	gi|55376769 protein Haloarcula marismortui	45.3	117	68	418	546	658	2.00E-22	108
**contig15246**	gi|91793955 Shewanella denitrificans OS217	47.37	76	234	10	565	639	2.00E-13	78.6
**contig13222**	gi|110637643 Cytophaga hutchinsonii ATCC	67.09	79	227	3	240	316	8.00E-21	103
**contig16115**	gi|269963230 Vibrio harveyi	47.76	67	1	198	233	299	5.00E-08	60.8
**contig04127**	gi|256396581 Catenulispora acidiphila DSM 44928	48.1	79	1	237	217	293	1.00E-15	86.3
**contig14791**	gi|19262983 Pseudomonas sp	45.61	57	201	31	27	82	3.00E-06	55.1
**contig20202**	gi|259488404 Aspergillus nidulans	62.16	37	117	227	103	136	0.001	46.6
**contig01911**	gi|119502900 marine gamma proteobacterium	53.62	69	80	286	591	659	2.00E-12	71.2
**contig22348**	gi|90021040 Saccharophagus degradans 2 40	59.57	47	142	2	1484	1530	2.00E-08	62
**contig25525**	gi|120436187 Gramella forsetii KT0803	49.21	126	379	2	2267	2392	1.00E-26	122
**contig07601**	gi|283844144 Bacillus cellulosilyticus	38.04	92	249	7	1	89	9.00E-07	56.6
**contig16702**	gi|53803512 Methylococcus capsulatus	36.77	155	30	488	104	254	2.00E-17	92.8
**contig18707**	gi|239820612 Variovorax paradoxus	71.88	32	57	152	39	70	2.00E-05	52.4

Supplementary Table 2. *Cont.*

Assembled contig ID	NCBI Accession number and Organism	% identity	alignment length	q. start	q. end	s. start	s. end	e-value	bit score
**contig18663**	gi|83950901 Roseovarius nubinhibens	59.72	72	2	217	318	389	7.00E-12	73.6
**contig25920**	gi|115372711 Stigmatella aurantiaca	44.29	70	203	3	2165	2234	3.00E-07	58.2
**contig22036**	gi|220928668 Clostridium cellulolyticum	54.17	48	177	34	1047	1094	7.00E-10	67
**contig23021**	gi|90022139 Saccharophagus degradans 2 40	54.55	33	3	101	379	411	0.001	46.6
**contig04598**	gi|228473605 Capnocytophaga gingivalis	30.57	193	555	40	35	220	5.00E-12	77
**contig10181**	gi|120436187 Gramella forsetii KT0803	44.17	120	2	361	2209	2328	7.00E-22	106
**contig11249**	gi|90022645 Saccharophagus degradans 2 40	50	34	5	106	706	739	0.014	42.7
**contig07930**	gi|198274843 Bacteroides plebeius	75	120	2	361	41	158	6.00E-51	203
**contig20761**	gi|254784408 Teredinibacter turnerae T7901	63.33	30	52	141	297	326	0.002	45.8
**contig25459**	gi|189347360 Chlorobium limicola DSM 245	46.99	83	1	228	1854	1936	4.00E-11	71.2
**contig20414**	gi|156740923 Roseiflexus castenholzii DSM	65.57	61	1	183	708	768	1.00E-08	63.2
**contig15701**	gi|90021040 Saccharophagus degradans 2 40	51.72	58	212	54	678	735	2.00E-08	62
**contig25008**	gi|153872900 cyclase Beggiatoa sp	44.16	77	230	3	152	221	9.00E-07	56.6
**contig00570**	gi|146301709 Flavobacterium johnsoniae	38.67	75	318	94	3526	3587	2.00E-06	55.5
**contig15304**	gi|260454326 Streptomyces flavogriseus	67.86	28	2	85	277	304	0.005	44.3
**contig21246**	gi|71736991 putative Pseudomonas syringae	46.75	77	233	3	4944	5002	2.00E-10	68.6
**contig23584**	gi|269124685 Thermomonospora curvata	63.41	41	124	2	412	452	3.00E-06	54.7
**contig00357**	gi|182681111 Xylella fastidiosa	55.56	27	19	99	188	214	0.029	41.6
**contig08179**	gi|83646812 Hahella chejuensis	62.5	32	179	274	390	421	8.00E-11	49.3
**contig15332**	gi|146301068 Flavobacterium johnsoniae	50	54	196	35	749	802	8.00E-05	50.1
**contig16110**	gi|255258429 Sideroxydans lithotrophicus ES.1	69.23	39	173	57	734	772	1.00E-08	62.8
**contig16669**	gi|90021040 like Saccharophagus degradans 2 40	69.23	26	27	104	1274	1299	0.04	41.2
**contig17172**	gi|90023532 Saccharophagus degradans 2 40	44.26	61	185	3	90	150	3.00E-04	48.1
**contig06033**	gi|110637740 Cytophaga hutchinsonii |	37.65	170	521	12	468	631	1.00E-24	116
**contig04332**	gi|159897943 Herpetosiphon aurantiacus	51.52	33	103	5	226	258	0.067	40.4
**contig01518**	gi|71282619 Colwellia psychrerythraea 34H	64.1	78	2	235	508	585	2.00E-19	98.6

Supplementary Table 2. *Cont.*

Assembled contig ID	NCBI Accession number and Organism	% identity	alignment length	q. start	q. end	s. start	s. end	e-value	bit score
**contig11361**	gi|253573954 Paenibacillus sp	40.63	64	198	7	208	268	6.00E-05	50.4
**contig25454**	gi|254786337 Teredinibacter turnerae T7901	28.88	187	1	549	239	383	4.00E-11	71.6
**contig15581**	gi|186686820 Nostoc punctiforme	70	40	3	122	958	997	8.00E-08	60.1
**contig02861**	gi|110639943 Cytophaga hutchinsonii	76.67	30	282	193	350	379	8.00E-11	46.2

Supplementary Table 3. Summary of multiple assembled contigs providing similarities with the same protein in the constructed Glycoside Hydrolase database.

454 Sequencing contig	UniProt Accession number	Organism known to encode the protein	UniProt description
**contig03455**<br>**contig04013**<br>**contig08782**<br>**contig15223**<br>**contig06190**<br>**contig23338**<br>**contig01813**<br>**contig14858**<br>**contig01054**<br>**contig12768**<br>**contig25208**<br>**contig04598**<br>**contig00570**	Q11PI8_CYTH3	Cytophaga hutchinsonii (strain ATCC 33406 / NCIMB 9469)	Beta-glycosidase related protein GH8
**contig12578**<br>**contig05391**	A7V9B3_BACUN	Bacteroides uniformis ATCC 8492	Uncharacterised protein GH9
**contig14077**<br>**contig10093**	B3PEQ9_CELJU	Cellvibrio japonicus (strain Ueda107	Endo-1,4-beta-D-glucanase (Cel9B) GH9
**contig01403**<br>**contig07930**	B5JIL5_9BACT	Verrucomicrobiae bacterium DG1235	Cellulase GH5
**contig09617**<br>**contig13222**	B7AFY4_9BACE	Bacteroides eggerthii DSM 20697	Uncharacterised protein of GH8
**contig13913**<br>**contig05967**	C0INS7_9BACT	uncultured bacterium BLR10	Cellulase GH 9

Supplementary Table 3. *Cont.*

454 Sequencing contig	UniProt Accession number	Organism known to encode the protein	UniProt description
**contig14342**<br>**contig11080**	C1S930_9SPHI	Chitinophaga pinensis DSM 2588	Chitinase GH18
**contig07270**<br>**contig08179**	C1UVR8_9DELT	Haliangium ochraceum DSM 14365	Cellulase
**contig01065**<br>**contig00162**	Q11SW6_CYTH3	Cytophaga hutchinsonii (strain ATCC 33406 / NCIMB 9469)	Endoglucanase realted protein GH9
**contig02397**<br>**contig02925**	Q11UC0_CYTH3	Cytophaga hutchinsonii (strain ATCC 33406 / NCIMB 9469)	Endoglucanase related protein GH 5
**contig08470**<br>**contig17865**	Q11VF9_CYTH3	Cytophaga hutchinsonii (strain ATCC 33406 / NCIMB 9469)	Endoglucanase related protein GH 9
**contig11249**<br>**contig11361**	Q21GB9_SACD2	Saccharophagus degradans (strain 2-40 / ATCC 43961 / DSM 17024)	Endoglucanase GH5
**contig01678**<br>**contig07103**	Q21HS9_SACD2	Saccharophagus degradans (strain 2-40 / ATCC 43961 / DSM 17024)	Endoglucanase like protein GH5
**contig05603**<br>**contig04413**	Q21NA7_SACD2	Saccharophagus degradans (strain 2-40 / ATCC 43961 / DSM 17024)	b-glycosidase GH 9
**contig23584**<br>**contig15581**	Q245S3_TETTH	Tetrahymena thermophila SB210	Cellulase GH5
**contig05463**<br>**contig15332**	Q2SCY8_HAHCH	Hahella chejuensis (strain KCTC 2396)	Beta-glucanase/Beta-glucan synthetase GH16

Supplementary Table 3. *Cont.*

454 Sequencing contig	UniProt Accession number	Organism known to encode the protein	UniProt description
**contig02705**<br>**contig12854**	Q2SHX0_HAHCH	Hahella chejuensis (strain KCTC 2396)	Beta-glucanase/Beta-glucan synthetase GH16
**contig02917**<br>**contig04098**	Q308B9_9BACT	Uncultured bacterium	Endo-1,4-beta-D-glucanase GH9
**contig11483**<br>**contig00357**	Q3R055_XYLFA	Xylella fastidiosa Ann-1	Cellulase GH6
**contig04503**<br>**contig14537**	Q9X3P6_9FIRM	Caldicellulosiruptor sp. Tok7B.1	CelB GH5
**contig17293**<br>**contig09391**<br>**contig22348**<br>**contig03216**	B7RVI9_9GAMM	marine gamma proteobacterium HTCC2148	Uncharacterised protein GH16
**contig04687**<br>**contig22358**<br>**contig16794**	C5BPZ6_9GAMM	Teredinibacter turnerae T7901	GH16 protein
**contig06707**<br>**contig05872**<br>**contig10691**	Q11UJ1_CYTH3	Cytophaga hutchinsonii (strain ATCC 33406 / NCIMB 9469)	Endoglucanase GH9
**contig02702**<br>**contig20063**<br>**contig15246**	Q12KZ3_SHEDO	Shewanella denitrificans (strain OS217 / ATCC BAA-1090 / DSM 15013)	GH16

Supplementary Table 3. *Cont.*

**454 Sequencing contig**	**UniProt Accession number**	**Organism known to encode the protein**	**UniProt description**
**contig15633**<br>**contig18210**<br>**contig04801**	Q21F54_SACD2	Saccharophagus degradans (strain 2-40 / ATCC 43961 / DSM 17024)	Putative retaining b-glycosidase GH5
**contig23140**<br>**contig15044**<br>**contig11559**	Q2SFD8_HAHCH	Hahella chejuensis (strain KCTC 2396)	Endoglucanase GH5
**contig19349**<br>**contig10599**<br>**contig00744**<br>**contig03003**	A0Y0X8_9GAMM	Alteromonadales bacterium TW-7	GH16
**contig18893**<br>**contig16578**<br>**contig09275**<br>**contig25883**	Q47XT7_COLP3	Colwellia psychrerythraea (strain 34H / ATCC BAA-681)	GH16
**contig06721**<br>**contig04690**<br>**contig07415**<br>**contig09168**<br>**contig01223**	Q11NQ3_CYTH3	Cytophaga hutchinsonii (strain ATCC 33406 / NCIMB 9469)	GH8
**contig26022**<br>**contig25780**<br>**contig00382**<br>**contig17731**<br>**contig20414**<br>**contig01518**<br>**contig02861**	A7LS44_BACOV	Bacteroides ovatus ATCC 8483	GH16

Supplementary Table 3. *Cont.*

454 Sequencing contig	UniProt Accession number	Organism known to encode the protein	UniProt description
**contig05277**	Q11VQ4_CYTH3	Cytophaga hutchinsonii (strain ATCC 33406 / NCIMB 9469)	GH8
**contig26107**			
**contig25564**			
**contig04461**			
**contig04615**			
**contig23608**			
**contig07156**			
**contig20679**			
**contig06547**			
**contig00226**	Q11PI9_CYTH3	Cytophaga hutchinsonii (strain ATCC 33406 / NCIMB 9469)	Beta-glycosidase related protein GH8
**contig26301**			
**contig06009**			
**contig04779**			
**contig03397**			
**contig08385**			
**contig25770**			
**contig26116**			
**contig16934**	Q21KX4_SACD2	Saccharophagus degradans (strain 2-40 / ATCC 43961 / DSM 17024)	Retaining b-glycosidase GH16
**contig00765**			
**contig01984**			
**contig24905**			
**contig01703**			
**contig25649**			
**contig15196**			
**contig15701**			
**contig16669**			
